# Drought Resistance in Rice from Conventional to Molecular Breeding: A Review

**DOI:** 10.3390/ijms20143519

**Published:** 2019-07-18

**Authors:** Yusuff Oladosu, Mohd Y. Rafii, Chukwu Samuel, Arolu Fatai, Usman Magaji, Isiaka Kareem, Zarifth Shafika Kamarudin, Isma’ila Muhammad, Kazeem Kolapo

**Affiliations:** 1 Institute of Tropical Agriculture and Food Security, Universiti Putra Malaysia (UPM), Serdang 43400, Selangor, Malaysia; 2 Department of Crop Science, Faculty of Agriculture, Universiti Putra Malaysia (UPM), Serdang, 43400 UPM, Selangor, Malaysia; 3 Department of Agronomy, University of Ilorin, Ilorin, P.M.B. 1515, Nigeria

**Keywords:** abiotic stress, drought tolerance, transgenic, QTL, markers assisted selection, crop improvement

## Abstract

Drought is the leading threat to agricultural food production, especially in the cultivation of rice, a semi-aquatic plant. Drought tolerance is a complex quantitative trait with a complicated phenotype that affects different developmental stages in plants. The level of susceptibility or tolerance of rice to several drought conditions is coordinated by the action of different drought-responsive genes in relation with other stress components which stimulate signal transduction pathways. Interdisciplinary researchers have broken the complex mechanism of plant tolerance using various methods such as genetic engineering or marker-assisted selection to develop a new cultivar with improved drought resistance. The main objectives of this review were to highlight the current method of developing a durable drought-resistant rice variety through conventional breeding and the use of biotechnological tools and to comprehensively review the available information on drought-resistant genes, QTL analysis, gene transformation and marker-assisted selection. The response, indicators, causes, and adaptation processes to the drought stress were discussed in the review. Overall, this review provides a systemic glimpse of breeding methods from conventional to the latest innovation in molecular development of drought-tolerant rice variety. This information could serve as guidance for researchers and rice breeders.

## 1. Introduction

Rice is a major staple food consumed by more than one-third of the world’s population, providing up to 80% of the daily calories intake of a vast majority of the human population, especially in Asia [1]. However, rice is considered one of the most drought-susceptible plants due to its small root system, thin cuticular wax, and swift stomata closure [1]. In the quest to attain self-sufficiency in rice production by 2050, the development of high yielding rice variety with a high degree of tolerance and resistance to both biotic and abiotic stresses is a prerequisite [2]. The yield potential of 10 t/ha can be obtained, however, the average harvest by local farmers is between 4–5 t/ha [3]. This wide gap in yield is attributed to various environmental stresses. Environmental stresses are natural phenomena that affect crop productivity and sustainability in agriculture. Stresses can be due to biotic factors such as pest, insect and disease incidence or abiotic such as flooding, salinity, drought, high temperature, air pollution, mineral deficiency, adverse pH, heavy metal toxicity, among others. Among the abiotic factors, drought is one of the most devastating and it has been estimated that more than 50% of the world’s arable land will be affected by drought in the year 2050 [4].

Currently, there is a scarcity of fresh water, with plant accounting for 65% of global freshwater usage [5]. The optimum amount of water required for irrigation to produce 1 kg of rice was estimated at about 3000 litres. As a result of drought stress, yield loss can rise up to about 100% depending on the growth stage of the plant. Yield losses must be minimized in order to help poor rice farmers in developing countries and for food sustainability to cater for the growing human population [5]. Globally, the increase in drought severity coupled with lack of high-yielding genotypes that are suitable for cultivation under drought conditions are the most limiting factors responsible for low production of rice. Rice cultivation is seasonal due to a lack of appropriate rice cultivars and techniques. The decline in water supply due to the loss of valuable groundwater resources affects rice production. There is also a severe depletion of watersheds due to deforestation and soil erosion in hills and mountains, thus supporting the claim that most agricultural lands will be affected by extreme environmental fluctuations caused by global climate changes [6]. For plants, the possibilities of escaping the drought condition are virtually nonexistent due to their immobile nature. Severe drought stress can be detrimental to plant development at all stages. During the reproductive growth stage, the effects of water deficit can lead to male sterility and embryo abortion soon after pollination which causes low reproductive success for many plant species [7]. Therefore, understanding how plants respond to the stress becomes imperative and primary to developing plants that are resistant to such stress.

Plant growth and development is the product of genotype, environment and the interaction between the genotype (genetic potential) and the environment [8]. The development also depends on biochemical processes (e.g., photosynthesis) that is connected to the environmental factors. When the environmental condition is less than the optimum requirement, plants become stressed and this adversely affects their productivity, growth and development [1]. There are two types of drought conditions, classified as terminal and intermittent [9]. Terminal drought condition is caused by a lack or decrease in water available to plants, thus leading to severe drought stress and the resultant death of the plant. However, intermittent drought condition leads to distress in plant growth during the period of inadequate rainfall or irrigation, which occurs either once or at intervals during the planting seasons. Unlike terminal drought stress, intermittent drought conditions are not usually lethal. Drought tolerance or resistance mechanisms depend on the ability of plant survival in maintaining function under terminal and intermittent drought conditions.

Drought tolerance is defined as plant tolerance under the minimum level of moisture content in the cytoplasm when the water content constitutes ~23% or ~0.3 g of the fresh and dry tissue, respectively. Drought tolerance mechanisms include cellular adjustments, physiological acclimation and morphological adaptations which are controlled by genetic factors at different stages. Cellular adjustments for drought tolerance involve increased chlorophyll content, lower osmotic potential and increase in harvest index. Physiological acclimation comprises higher stomatal density and conductance; decreased transpiration rates; reduced and early asynchrony between female and male flowering and maturation; and better production, accumulation, assimilation, and seed and biomass yield partitioning. On the other hand, morphological adaptations include increased root thickness and length, waxy or/and thick leaf coverings, decreased leaf weight and size, smaller epithelial cells, delayed leaf senescence and increased green leaf area [1].

The increasing occurrence and significance of drought in crop production have made it an integral subject of research over the past few decades. Nevertheless, the quantitative and complex nature of the drought-tolerant trait has made it challenging to study drought responses. In analysing drought response in plants, the stage, severity, timing and mode of the drought stress and its occurrence with other abiotic factors such as temperature and salt stresses are significant [10]. Moreover, different cultivars, subspecies and species of crops have shown genetic variation in response to drought tolerance under the same environmental conditions, highlighting the importance of diversity as a principal factor in drought tolerance and its importance in drought-related studies. The cultivars that exhibit high drought tolerance are often targeted for drought-related research and are the most promising sources of drought-related genes to be used in the development of modern crop varieties. Therefore, understanding the way plants respond to drought stress is one of the most important steps in the development of the drought-tolerant crop. The objectives of this review were (i) to describe the current method of developing a durable rice drought resistant variety through conventional breeding and the use of biotechnological tools and (ii) to comprehensively review the available information on drought-resistant genes, QTL analysis, gene transformation and marker-assisted selection.

## 2. Drought Stress: Perception, Biochemical Responses and Mechanism

The term stress is often defined physiologically, as a response to different situations. Generally, stress is an alteration of the physiological conditions, triggered by factors that tend to disrupt the stability of the plant [4]. The regular fluctuation in environmental factors as a result of predictable circumstances on daily and seasonal cycles allows plant to adapt to regular changes in their metabolism. Therefore, every deviation from the optimum condition does not necessarily lead to stress [4]. Stress is a highly unpredictable fluctuation or constriction imposed on normal metabolism that causes disease, injury or aberrant physiology. Plants are regularly exposed to various stresses while growing in their natural habitat. Drought is a climatic factor characterized by low or lack of rainfall. Mostly, drought stresses occur when there is a low level of water in the soil and a continuous loss of water through evaporation and transpiration.

**Figure 1:**
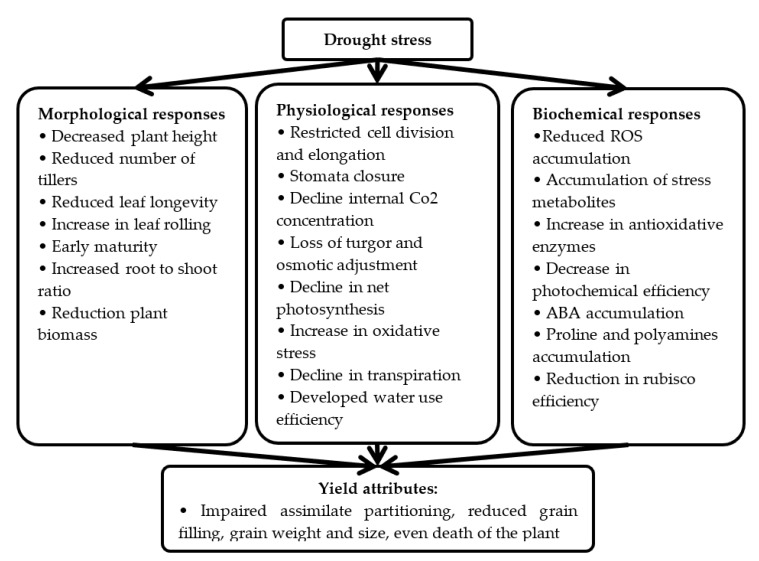
Annotation mechanisms of growth/yield decline in plants under drought stress conditions.

Generally, drought stress is measured by morphological, physiological and biochemical response which are characterized by a reduction in plant water content, a decrease in cell elongation and growth, closure of stomata, reduction in gaseous exchange, and disruption of enzyme-catalyzed reactions (Figure 1). Moreover, under severe drought conditions, there is a gross disruption in photosynthesis and metabolism which eventually leads to the death of the plant [7]. According to Anjum et al. [11], drought stress hinders cell enlargement as compared to cell divisions. This hindrance in plant growth affects different biochemical and physiological processes including ion uptake, respiration, photosynthesis, growth promoters, carbohydrate, source–sink relationship, and nutrient metabolism [12]. In plants, understanding the physiological adaptation to changes in drought resistance could be used as selection criteria for the development of high yielding variety under drought conditions.

In order for drought resistance mechanisms to be activated, plant cells must sense an above or below ground incidence of an imbalance between water loss and water availability, after which that perception is converted into a cellular stress signal. As sessile organisms, plants have evolved a complex signalling network that conveys stress messages throughout the plant via multiple primary and secondary signalling transduction pathways. These pathways consist of various types of signalling molecules since a combination of hormone signals coupled with the accumulation of other metabolic compounds such as reactive oxygen species, proteins, and other osmolytes are often required for changes in gene expression. These compounds may be either actively produced by the plant or accumulated as a result of cellular damage [10]. The signalling cascades that occur may be either the cause of and/or are in response to the perception of drought stress to actively initiate further downstream changes in gene expression leading to plant drought resistance and are initiated by plant hormone signalling pathways.

Reduction in osmotic potential in the cytosol is a result of the accumulation of organic and inorganic solutes, which leads to the maintenance of turgor pressure under drought conditions. This biochemical procedure is a type of osmotic adaptation that strongly depends on the water stress level. Osmotic adaptation occurs via the accumulation of proline, sucrose, glycine betaine, and other solutes in the cytoplasm, promoting water uptake. Proline is the most widely investigated in osmotic adaptation due to its considerable stress reducing ability under adverse conditions [13]. Proline acts as an osmolyte in plants under various adverse conditions [13]. The differences in proline accumulation under normal and stress conditions have been reported in rice [19,20,21].

Antioxidants are vital reactive oxygen species (ROS) scavenging components in crops, and their expression increases drought tolerance in rice [14]. The imbalance between the generation and quenching of ROS is the most common phenomenon under drought stress [15]. The ROS include hydroxyl free radicals, superoxide radical hydrogen peroxide and singlet oxygen, and they cause protein denaturation, lipid peroxidation, disruption in cellular homeostasis, cellular oxidative damage and DNA mutations. A complex antioxidant system containing enzymatic antioxidants and non-enzymatic molecules protects plants against the adverse effect of ROS. Enzymatic antioxidants include monodehydroascorbate reductase (MDHAR), dehydroascorbate reductase (DHAR), superoxide dismutase (SOD), catalase (CAT), glutathione reductase (GR), ascorbate peroxidase (APX), guaiacol peroxidase (GPX), and ascorbate-glutathione cycle enzyme [1], while ascorbate (AsA) and glutathione (GSH) serve as non-enzymatic antioxidants within the cell. Increase in levels of drought stress triggers the increase in enzymatic and non-enzymatic antioxidants activities in rice [15]. The trend for these antioxidant defence enzymes to increase their activity demonstrates their protective activity in neutralizing the oxidative injury induced by drought stress.

Polyamines (PAs) are small positively charged molecules that act in response to drought stress in rice [16]. The PAs in plants include putrescine (Put), spermidine (Spd) and spermine (Spm). It can interact with different signalling networks and also regulate stabilize membranes, osmotic potential ionic and homeostasis. The PA content increase during drought stress is directly correlated with increased photosynthetic capacity, decreased water loss and improved osmotic detoxification and adjustment. The roles of PAs consist of regulating gene expression via maintenance of ion balance, facilitating the DNA binding of transcription factors, stabilizing membranes, scavenging radicals, and preventing senescence via the conformational transition of DNA and protein phosphorylation [1]. A recent study on rice demonstrates that rice produces much higher levels of putrescine under stress, promoting spermidine and spermine synthesis and ultimately protecting the plants from drought [16].

Major plant hormones that are important in drought response are abscisic acid (ABA), cytokinins (CK), Jasmonic acid, ethylene, and others. Drought stress is perceived as a hydraulic pull caused by soil to plant gradient of pressure due to soil drying. When a hydraulic pull is sensed, the result is a shift in the concentration of the signal hormones ABA [17]. ABA concentration typically increases in order to convey the drought stress signals, whereas other hormones such as CKs may be reduced by downregulation of gene expression, degraded by oxidase enzymes activity, or due to stress damage. These changes are complex and dynamic since hormone concentration may act independently to confer a signal or it may act in conjunction with other hormones and/or with other signals [17]. Furthermore, the endogenous concentration of a given hormone may be influenced by the duration and severity of drought stress and may differ in different plant organs. For instance, hormones working in conjunction with each other are exemplified by the indirect role of ABA in water stress signalling by inhibiting the synthesis of ethylene [18]. ABA-dependent and ABA-independent signalling pathways are used to elicit a response to drought and a rapid accumulation of ABA has been correlated with enhanced drought resistance [1]. In studies of the highly drought tolerant resurrection plants (*Craterostigma wilmsii*), ABA concentrations were shown to be the most highly affected hormone in response to drought stress [17]. ABA and other hormonal signalling pathways lead to major changes in plant growth, defence responses, and major drought tolerance mechanisms.

**Figure 2:**
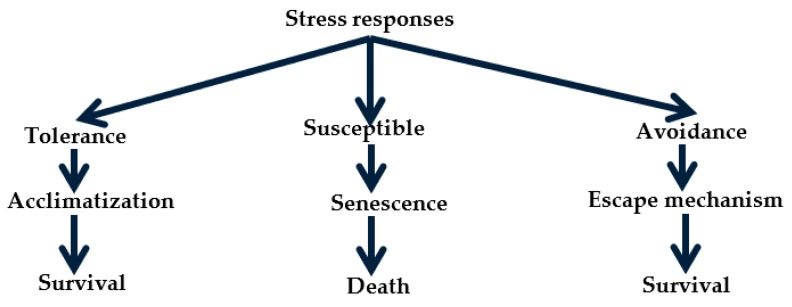
Plant response mechanisms to drought stress.

There are several literature works on drought resistance with different definitions which are fundamentally coined to explain plant adaptation mechanisms to stress defence, water productivity, drought resilience, drought avoidance, drought survival, stress protection, drought adaptation, drought tolerance and drought homeostasis. Basically, drought stress is on three-dimensional axes which include drought severity, duration and timing. Drought tolerance can be referred to as the propensity of the plant to grow, develop and turn out good economic yield under water stress [19]. According to Chourasia [20], drought tolerance was classified based on water potential of plant tissue. Drought tolerance is the plant ability to maintain plant tissue under high water potential and avoid the effect of water stress. Meanwhile, at low tissue water potential, drought tolerance enables the plants to sustain water deficits so as to maintain their metabolic function when tissue water potential is significantly low [7]. Plants typically utilize various resistance mechanisms which are categorized as the escape, avoidance, or tolerance response to drought stress. Escape is typically characterized by a state of dormancy or necrosis caused by programmed death of cellular tissues, with the exception of those that are required for regeneration or regrowth upon a change of season or alleviation of stress such as renewal of available water after prolonged drought conditions. Drought avoidance responses involve a group of mechanisms including deep rooting or leaf curling to prevent, reduce, or delay cellular dehydration. According to Luo [21], plant response to drought is classified into tolerance and avoidance mechanism (Figure 2).

## 3. Conventional Breeding

Conventional breeding methods include induced mutation, inter-generic and inter-specific crosses. The recent advancement in plant physiology has led to improved techniques and tools that help in the development of drought tolerance. Due to low heritability and the large influence of genotype by environment interaction, grain yield is used as the selection criteria for superior cultivar under drought conditions; however, this has been proven to be ineffective [22]. Over time, the focus of conventional breeding has shifted to selection based on physiological characters since these traits are less time-consuming and dependent on genetic variation. The major problem of abiotic stress tolerance is the complexity resulting from several factors such as the lack of effective selection criteria, low genetic variability under drought condition for yield and yield component, complication between stress parameter and various biochemical, physiological and molecular phenomena that are affecting plant development and growth [23]. However, high yielding variety under well water condition is the major target in crop breeding, and, in many cases, high yielding variety can still give high to moderate yield under drought conditions [24]. Basically, conventional breeding methods are important for germplasm conservation, hybridization between sexually distinct parent, and development of novel genetic traits. Over the past three decades, the international rice research institute (IRRI) has developed a vast range of elite cultivars that are resistant to different diseases and abiotic stresses using conventional breeding techniques [25]. Recently, pedigree selection, recurrent selection, backcrossing, and induced mutation have become the major methods used in conventional breeding.

**Figure 3:**
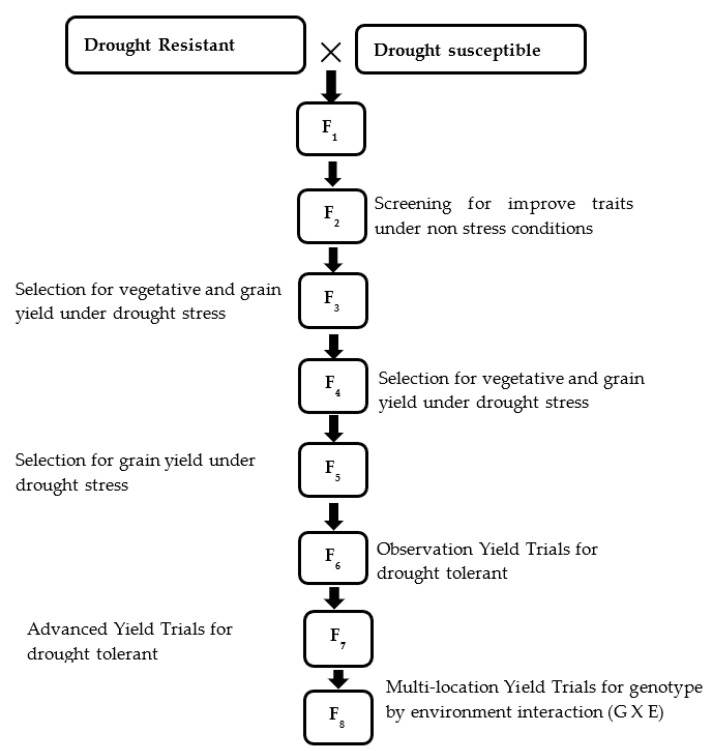
Modified method for conventional yield trail in rice.

The pedigree selection is one of the oldest and most widely used breeding methods in rice improvement. This method is highly appropriate for developing resistance in rice especially if the trait is governed by major genes. One of the major advantages of pedigree selection is that the combination of many genes controlling biotic and abiotic can be achieved [25]. However, the major disadvantage of pedigree selection is that it is time-consuming and requires an evaluation of many lines periodically all over the planting seasons, while keeping a record on selection criteria. Among the breeding methods, pedigree selection requires high familiarity with the breeding materials and also the influence of genotype by environment on traits of interest. This method is not suitable for the trait under the influence of many genes; in this case, the diallel mating design will be suitable for selection [25]. Generally, in most self-pollinating crops including rice, plant breeders prefer recurrent selection over pedigree selection [3]. The overall selection procedure for the development of drought tolerant in rice was presented in Figure 3.

Recurrent selection is used in varietal improvement which involves multiple crosses to gather favourable alleles while still maintaining the genetic diversity. It provides shorter and defined breeding cycles, more precise genetic gains, and development of highly diverse breeding lines. This method has been widely studied in maize [22], rice [26] and soybean [27]. In wheat, this method has been successfully used for the improvement of grain yield [28], kernel weight [29] and the percentage of grain protein [30]. In summary, the efficacy of this method with respect to improved agronomic traits and enhancement of drought tolerance has shown that this method is superior to the pedigree selection method.

The conventional backcrossing technique is commonly used in rice breeding for introgression of desirable or target gene controlling a particular trait from donor parent to recipient parent with the aim of reducing the genome of the donor parent and subsequently increasing high recovery of recipient parent. This technique provides an accurate and precise way of developing a large number of advanced breeding lines [31]. The use of backcrossing methods has led to the development of drought-tolerant varieties in rice [31].

Induced mutation is another breeding technique used to complement conventional breeding methods, since the method has been proven to be effective through the development of improved agronomic traits such as an increase in grain yield [32], resistance to pests and diseases, and improvement of physical grain quality [33]. The conventional plant mutation breeding is used in the development of a new variety(ies), the major advantage of induced mutation is the creation of gene alleles that are not found in nature. The new gene alleles created in the new variety can either be used directly as a commercial cultivar or in a breeding program. Oladosu et al. [34] summarized the use of induced mutation with many success stories on groundbreaking rice varieties developed through induced mutation. In Myanmar, the Manawthukha rice variety was irradiated with a dose of 300 Gy of gamma rays from _60_Co source to screen for drought tolerance by withdrawing irrigation after 90 days of transplant until harvesting. After sixth generations of evaluation and selection, two mutant lines namely: MK-D-2 and MK-D-3 were selected as drought tolerant using physiological screening techniques [35]. Similarly, an Iranian rice landrace “Tarom Mahalli” was irradiated with gamma source using an optimum dose of 230 Gy, at M_4_ generation, 11 lines were selected with drought-tolerant characteristics [36]. A super green rice mutant that is high yielding and drought tolerant under low fertilizer-water efficient and water deficiency was developed through induced mutation in Indonesia [37]. In Malaysia, two superior lines MR219-9 and MR219-4 having high yield potential and drought tolerant character were derived from popular MR219 rice variety [38]. Until today, this technique has continuously been used by plant breeders for generating variation and development of new traits in rice improvement.

With the initiation of advanced approaches in biotechnology, our knowledge about plant responses to drought at the whole plant and molecular level has increased rapidly. Hundreds of genes expressed under drought stress have been identified and some have been cloned. Different methods such as transgenic and gene expression pattern are generally employed in drought tolerance development. New methods, such as proteomics, genome-wide association, stable isotopes, and fluorescence or thermal imaging, have contributed to bridging the gap between genotype–phenotype. The major tools in biotechnological methods are molecular technology and genetic engineering which has led to the development of drought tolerance in rice. Generally, development of genetic resistance is the preeminent and stable approache to reduce the effect of drought.

## 4. Biotechnological and Molecular Approaches for Drought Tolerance

The mechanism of drought tolerance is complex due to variations in plant phenology; moreover, drought traits are controlled by multiple quantitative trait loci (QTLs) [39]. Plant response is complex and difficult to understand unless a thorough study of the physiological and genetic bases is conducted. Neither the traditional breeding techniques nor modern genetics can effectively improve the drought tolerance of crop plants if the molecular mechanisms correlating with the grain yield stability are not adequately considered [40]. Advances in plant physiology, phenotyping, and plant genomics have led to new discoveries in crop drought tolerance. Hence, crop breeders will be able to increase crop yields using the latest gene discovery for plant improvement [5]. As improving plant physiology enhances our knowledge on the complexity of drought-tolerant mechanism and its relation with different traits, selection efficiency using molecular and genomics approaches will result in the identification of QTLs and genes linked with traits. The identification of the candidate genes responsible for plant tolerance under different abiotic stress is essential to developing transgenic crops with enhanced drought stress tolerance [5]. Once the gene controlling drought tolerance has been identified through QTL mapping, these genes can then be incorporated into the genetic background of any desirable cultivar using genetic engineering (*Agrobacterium tumefaciens* or gene gun) and hybridization using marker assisted selection.

### 4.1. QTL for Drought (List of QTL Genes for Drought)

Basically, identification of QTL controlling particular traits under drought stress involves a chain of activity such as mapping populations in which the traits of interest correlating with drought tolerance are segregated; identification of polymorphic markers; genotyping the mapping populations using polymorphic markers; construction of genetic maps; accurate phenotyping based on drought tolerance-correlated traits; and, lastly, QTL mapping according to genotypic as well as phenotypic data. Several linkage mapping studies conducted on drought tolerance in different crops have been reviewed by Sahebi et al. [1]. Due to limitations associated with mapping populations such as inheritability and genotype by environment interaction, linkage analysis-based QTL mapping cannot offer detailed information about QTLs. These limitations include the following: the segregation of different QTLs linked to the same traits in diverse mapping populations, inadequate phenotypic variation related to existing traits in the mapping population, and, lastly, the identified QTLs are commonly associated with large chromosomal segments or genomic regions due to insufficient time for recombination [41]. Linkage disequilibrium based on association mapping used in human genetics has been proposed as an alternative to QTL mapping in order to overcome some of the limitations in various crop species [42]. The association mapping technique entails five steps: (a) selection of various individual groups or panels from a natural population pool; (b) accurate records of phenotypic data on each group; (c) high-density sequencing of interesting candidate genes or the panel’s genotyping markers; (d) studying the level of genetic differentiation among groups within the particular population (population structure) and the relatedness coefficient between individual pairs within the population (kinship); and (e) analyzing the association mapping according to data obtained on population structure, kinship, and the correlation of genotypic and phenotypic data [1].

The advantages of the association mapping over biparental linkage mapping include higher resolution due to the utilization of all recombination events throughout the evolutionary history of a specific crop species; bypassing the development of a particular mapping population and the provision of a natural germplasm collection for a specific crop to reduce the required time for QTL mapping; using the same genotyping data and association mapping group for mapping different traits makes it cost-effective; eliminating randomly recombinant inbred lines that express an insufficient agronomic type from the population’s structure; and being able to sample and present many alleles per locus relative to linkage mapping (a survey of only two alleles) [42]. In summary, many QTLs related to drought tolerance in rice have been identified (Table 1). However, only a few studies on grain yield have been reported. Most QTLs in rice have been identified based on a wide range of important traits, including root and shoot responses, osmotic adjustment, hormonal responses, photosynthesis and whole plant response to drought tolerance.

### 4.2. Genetic Engineering for Drought

Plants have evolved consistent pathways or signalling chain process for stresses by producing different classes of protein which includes transcriptional factors, molecular chaperones, enzymes and other functional proteins [65]. These proteins enhance plant resistance or tolerance to drought conditions. In fact, hundreds or even thousands of these genes (regulatory element and protein) have been identified using different genomic approaches. In rice, these genes have been incorporated into the rice genome to study their effect on drought improvement either by suppression or overexpression as indicated in Table 2.

In rice, different biological processes are controlled by different transcription factors encoded by *WRKY* genes. Zinc finger proteins, particularly those that regulate stress responses, are widely distributed in plants. The *WRKY* genes are broadly distributed among plants and are present in monocotyledons and dicotyledons. Many *WRKY* genes play positive or negative regulatory roles in plant responses to different abiotic stresses [1]. For example, rice zinc-finger protein (dst mutant) showed improved drought and salt tolerance by reducing stomata density and increasing stomata closure. However, DST non-mutants act negatively on stomata closure by modifying H_2_O_2_ homeostasis [66]. Overexpression of Zinc finger proteins *OsZFP252* showed 74–79% higher chances of survival by enhancing drought tolerance. It also increases soluble sugar and proline accumulation [67].

During drought conditions, plants synthesize and accumulate abscisic acid (ABA) in the guard cells to help activate closure of the stomata in order to reduce the amount of water lost [68]. Some ABA genes have been identified as drought tolerant in different crops [10]. When *LOS5/ABA3*, a major enzyme in the final stage of ABA biosynthesis, was overexpressed in transgenic rice, it was observed that the grain filling and grain yield was improved under drought stress conditions [10].

The late embryogenesis abundant (*LEA*) proteins are primarily found in plants and it covers a number of intrinsically unstructured proteins (IUPs). These small proteins ranging from 10 to 30 kDa are formed during the maturation drying process of embryo development and they act as chaperones [69]. Drought produces a cellular water deficit and leads to the accumulation of *LEA* proteins. In plants, a number of reports indicated that over-expression of *LEA* proteins from various groups confer tolerance on a variety of water deficit treatments [70]. Over-expressing *OsLEA3* in rice enhanced drought tolerance in the field response to water deficit stress [71]. When the *HVA1* (gene encoding *LEA* protein) from barley was overexpressed on wheat and rice, there was a significant increase in growth performance and water-use efficiency under drought stress [72,73]. The encoding *LEA* gene *OSLEA3-1* in rice was reported to play a significant role in regulating drought stress [71]. Recently, overexpression of *OsLEA3-2* in rice also showed a similar trend of drought tolerance, and the yield loss was less compared to control treatment under a severe drought field condition [70].

The structural integrity of membranes could be maintained by accumulation of osmolytes; many previous studies have reported that plants were more tolerant to water stress which favour osmotic adjustment [74]. Osmotic adjustment is known to be a part of the water stress avoidance mechanism. Proline acts as an osmolyte in plants under various adverse conditions [13]. The differences in proline accumulation under normal and stress conditions have been reported in rice [15]. Additionally, proline exhibits three main roles under stress, i.e., proline can act as a signalling molecule, an antioxidative defence molecule, and a metal chelator [13]. Under drought stress, the accumulation of this amino acid might repair damage by increasing the rate of antioxidant activity [14]. In addition, proline is also known as a reactive oxygen species (ROS) scavenger and prevents oxidative damage. Maize plants subjected to drought stress increases its proline in order to maintain osmotic adjustment [75]. It was reported that the intensity of proline accumulation was dependent on the degree of water reduction and plant species [74]. Thus, the proline content can be used as a marker for screening drought tolerance in rice. In transgenic rice, overexpression of a proline gene enzyme-encoding 1-pyrroline-5- carboxylate synthetase (P5CS) showed a great improvement for drought tolerance [76]. Similarly, overexpressing *OsOAT* gene increased the level of proline and improved drought tolerance and oxidative stresses [77].

Trehalose, also known as tremalose or mycose, plays a significant role in abiotic stress such as cold and drought. It stabilizes proteins against denaturation, acts in stress protection and also reserves carbohydrates. In plants, biosynthesis of trehalose is catalyzed by two main enzymes, namely, trehalose-6-phosphate synthase (TPS) and trehalose-6-phosphate phosphatase (TPP); accumulation of trehahole in rice has been reported to improve drought tolerance. When a fusion TPP/TPS gene derived from Escherichia coli (*otsA* and *otsB*) was engineered into rice, the results showed an increase in trehalose, improvement in drought tolerance and the rice plant exhibiting less photooxidation in cold and salt stress [78]. In summary, this report suggested that engineering of drought tolerant genes into the genetic background of rice is promising, provided that a drought-inducible promoter is used to obtain effective results.

### 4.3. Marker-Assisted Selection (MAS) for Drought Tolerance

MAS is a DNA based marker employed by plant breeders for three main purposes: (a) to accumulate favourable alleles by tracing the desirable alleles as either dominant or recessive through generations, (b) to identify desirable individuals from segregated breeding lines based on entire genome or part of allelic composition, and (c) to introgress favourable alleles by breaking the undesirable linkage loci. The general terms used in modern breeding methods include marker-assisted selection (MAS), marker-assisted pedigree selection (MAPS), genomic selection (GS) or genome-wide selection (GWS), marker-assisted recurrent selection (MARS) and marker-assisted backcrossing (MABC). From the methods listed above, MABC is the most effective and widely used method [101,102,103]. When a gene is responsible for a high percentage of phenotypic traits or the expression of a desirable trait is controlled by a single gene, then the transfer of a specific region from a donor parent to the recipient parent can show a significant improvement in such trait using MABC.

The use of MABC has been employed by many plant breeders due to its relative cost-effectiveness and timeliness as compared to the conventional phenotypic methods. The use of MAS in backcrossing (BC) has three major advantages: (a) for difficult phenotypic traits, selection of a linked maker to an allele from a donor cultivar at a locus closer to the target gene of interest can increase the accuracy and effectiveness of selection; (b) it enables introgression of a desirable gene from a donor parent to a recipient parent while maintaining the essential characteristics of the recipient parent; (c) when transferring recessive gene using conventional breeding methods, additional selfings are required in each backcross generations, leading to a practice that is excessively low for most breeding purposes; this can be overcome through MAS. Therefore, MAS has been proven to be an advance strategy for increasing selection gain, particularly in phenotypic selection. Moreover, according to Knapp [104] and Lande and Thompson [105], the theory of quantitative genetics suggests that the efficacy of MAS is inversely proportional to the heritability of the desirable traits. The theory developed by Knapp [104] for estimating the selection of one or more genotypes using MAS defines the parameter for estimation of the cost-effectiveness of MAS in relation to phenotypic selection. It is estimated that plant breeders using conventional phenotypic selection must test at least 1.0 to 16.7 times additional breeding lines compared to breeders using MAS to be guaranteed the selection of one or more superior genotypes depending on the traits’ heritability, selection pressure, and genotypic superiority. Hence, MAS can significantly decrease the time and resources required to accomplish a selection goal for heritability traits of low to moderate values when the selection intensity is high.

**Figure 4:**
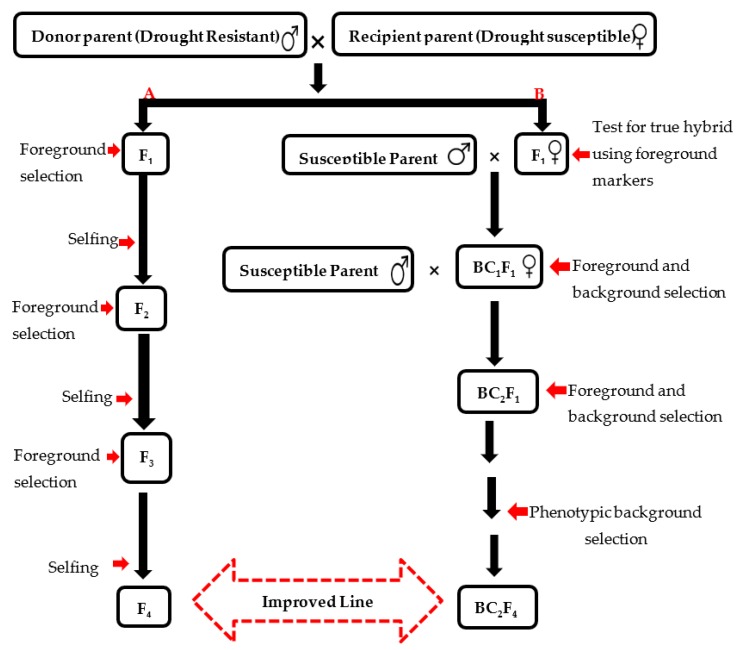
Method of development of drought tolerance in rice through (**a**) marker-assisted pedigree selection; (**b**) marker-assisted backcrossing.

MAS offers the most accurate, environmentally-friendly, fast and economical method of developing superior rice varieties with a certain degree of resistance or tolerance to drought. Countless QTL have been identified, cloned and even introgression in a rice genetic background using genetic engineering tools for the development of drought-resistant rice varieties. Analytical and theoretical studies have revealed that the maximum selection effectiveness for quantitative characters can be obtained through a combination of phenotypic and molecular information. Therefore, DNA markers provide the gateway for the identification of superior genotypes in early generations and hence have a solid impact on breeding programs by decreasing the number of progenies evaluated and, as such, hastening the breeding circles [106]. An overview of the approach used in developing drought tolerant rice varieties through marker-assisted backcrossing and pedigree selection was presented in Figure 4.

## 5. Limitations and Future Prospects

Conventional screening of improved drought tolerance genotypes still relies on the manual process. This process is error-prone, labour intensive, time-consuming, often very inefficient and involves destructive sampling [107]. All of this makes the conventional method relatively inefficient compared to the improved genetic techniques. To overcome the limitation in genotype–phenotype studies, more reliable and effective, automated, multifunctional, and high-throughput phenotyping has been developed and is proficient in screening multiple genotypes concurrently. Furthermore, plant phenomics helps in bridging the gap between phenotyping and high-throughput methods for identification of markers [108], thus helping to fast-track plant breeding targeted at increasing yield and the resistance to biotic and abiotic stresses [107]. Phenomics consist of an integrated plant physiology, linking with genomics through the advancement made in image analysis, machine vision, robotics, and computing to widen the scope of plant biology. The transdisciplinary approach employed in plant phenomics cuts across mathematics, physics, and biology, rather than biochemistry, genetics, plant breeding and physiology. Several phenomics studies have been reported in rice salinity stress [109], detection of QTL drought tolerance in wild barley [110] and responses of a C4 crop plant to water and nitrogen deficiency [111]. Although the report on drought response of different crops has been well documented, there are limited studies on identification of genotypic variation on water-use efficiency and drought tolerance in rice.

Similarly, the advent of genome editing technology has outmoded the traditional breeding methods’ limitations, and this marks the beginning of a new genomic era in crop improvement. Genome editing involves the use of site-specific nucleases (SSNs) engineered to modify target genes at a desirable location on the genome. The SSNs breaks the double-stranded DNA at a target location through the use of clustered regularly interspaced short palindromic repeats associated endonuclease Cas9 (CRISPR/Cas9), transcriptional activator-like effector nucleases (TALENs) and zinc finger nucleases (ZFNs). The double-stranded DNA break is then subjected to a natural repair mechanism either through homologous recombination or non-homologous end joining [112]. According to Bortesi and Fischer [113], the non-homologous end joining repair pathway is error-prone, thus resulting in the knockouts of targeted gene and frameshift mutations due to deletions and insertions (indels). However, the homologous recombination pathway is more accurate in exchange for a homologous sequence leading to gene replacement or gene knockout [114]. CRISPR/Cas9 is the most advanced genome editing technology in plant breeding [115]. This system has been used successfully in major food crops due to its high precision, adaptability and simplicity [116]. Shim et al. [117] reported that overexpression of OsNAC14 made the rice plant impenetrable to drought during the vegetative stage. Similarly, field evaluation revealed that overexpression of OsNAC14 transgenic rice lines led to a higher filling rate and increased the number of panicles compared to the non-transgenic plants, when subjected to drought conditions. In this study, it was demonstrated that CRISPR-Cas9 induced OsNAC14 specifically regulates the expression of OsRAD51A1 and regulates other downstream target genes for defense-related DNA repair, strigolactone biosynthesis, and stress response which together confer drought tolerance in rice.

Lastly, inadequate phenotyping screening of newly developed drought-resistant genotype mimicking the exact field conditions is a major setback in drawing conclusions on the effectiveness of discovered genes in relation to drought resistance. Despite the major breakthrough in gene discovery through QTL mapping, genetic engineering and marker-assisted selection, the true picture of events, as seen from the condition of local farmer harvest, is only a small fraction of the acclaimed achievement. The reality on the ground among local farmers in affected areas is still disproportionally minor to all the achievements. It is obvious that the inconsistency between the resources’ channels into the gene discovery research program and the research outcome is not solving the current problem of drought stress. Several research works have recognized this problem and have attempted to define a better approach either directly or indirectly by developing field screening techniques to enhance drought resistance towards improved productivity. The correct protocol for drought stress evaluation involves stress peak, timing and duration within a target environment or having a broad spectrum of drought stress. In most literature on drought, plant breeders and physiologist tend towards field evaluation for phenotyping studies while molecular biologists are more restricted to glasshouse or growth chamber evaluations. Volaire et al. [118] argued that the correct protocol for drought tolerance can only be understood from the point of ecology, physiology and breeding of individual plant species. Blum [119] outlined some basic requirements to be considered in drought evaluations. However, these suggestions do not rule out other protocol or relevant tests:

1. It is highly questionable that only one experiment in the laboratory determines the ability of a certain gene to confer drought resistance that is beneficial in breeding.
2. Traits of interest should consist of aerial and root organs, or phenology, particularly those influencing plant–water relations. This is expected to help in the setup of succeeding stress trials and drawing a conclusion on the transgene.
3. Multiple stress trials should be conducted to determine early whether transgenic plants have the ability to avoid and/or tolerate dehydration.
4. In general, the relative water content is preferred to leaf water potential as a measure of leaf water status since the relative water content also signifies the capacity for osmotic adjustment. For the same leaf water potential, a dehydration-avoidant genotype will express higher relative water content.
5. In the case where dehydration avoidance is detected in the transgenic plant, the trait should be evaluated to confirm whether it results from sensitive stomatal closure, turgor preservation by osmotic adjustment or preservation of turgor due to enhanced ability of the root to take up soil moisture.
6. Measurement of physiological functions should be associated with growth and photosynthesis. Antioxidant accumulation and function are also common measurements despite their relationship with whole-plant growth are not as well established, unlike photosynthesis.
7. Due to the effect of dehydration avoidance or tolerance of meristems on plant survival, survival or recovery from stress should, among others, be determined by relative water content at peak stress prior recovery.

## 6. Conclusions

The timing, endurance and strength of drought stress under natural conditions are dynamic and highly unpredictable, and this complicates evaluation for drought resistance. In addition, evaluation for drought resistance should be in association with related abiotic stresses such as salinity and high temperature because of their correlations with drought stress. Several QTLs for drought resistance have been identified in rice; subsequently, many attempts have been made using these QTLs. The recent advancement in functional genomics has made it possible to conduct high throughput genotyping which helps in identifying major QTL responsible for drought tolerance. Hence, the successful cloning of these QTL for drought traits will facilitate a better understanding of the genetic basis of drought resistance. However, the most concrete application of drought resistance QTLs is to perform marker-assisted selection based on pyramiding of favourable QTL alleles for development of drought-resistance in rice using the newly emerging breeding techniques such as GWS and MARS. Using transgenic approaches, many genes have been identified and used for improving drought tolerance; however, most of the research was conducted in the glasshouse. Therefore, owing to the complexity of field conditions, those genes that are proven to be drought resistant should be further tested on the field before use in the breeding program. Similarly, most studies on drought-resistance focus on the above ground traits leaving a great vacuum for below ground traits mainly due to difficulty in phenotyping. Therefore, root plasticity and architecture should be given adequate attention in drought-resistance study because they play major roles in growth and stomata regulation under drought conditions.

## Figures and Tables

**Table 1:** QTL for shoot and root responses under drought stress conditions.

Trait	Population	Marker	Type	QTL	References
Seedling drought resistance	Indica × Azucena	RFLP, AFLP & SSR	Recombinant inbred line	7	[43]
Cellular membrane stability	IR62266 × CT9993	RFLP, AFLP & SSR	Doubled haploid line	9	[44]
Leaf water relations and rolling	Azucena × Bala	RFLP, AFLP & SSR	Recombinant inbred line	13	[45]
Seed fertility, spikelet per panicle and grain yield	Teqing × Lemont	SNP	Introgression lines	5	[46]
Root number, thickness, length, and penetration index	IR58821 × IR52561	AFLP & RFLP	Recombinant inbred line	28	[47]
Root architecture and distribution	IR64 × Azucena	RFLP	Doubled haploid line	39	[48]
Root traits	IR1552 × Azucena	SSR	Recombinant inbred line	23	[49]
Deep roots	3 populations	SSR, SNP	Recombinant inbred line	6	[50]
Root penetration, root number, and tiller number	CO39 × Moroberekan	RFLP	Recombinant inbred line	39	[51]
Root-penetration	Azucena × Bala	RFLP & AFLP	Recombinant inbred line	18	[52]
Grain yield under drought	Two population	SSR	Bulk-segregant and Selective genotyping	-	[53]
Grain yield in aerobic environments	Three populations	SSR	Bulk-segregant	1	[54]
Yield and yield traits at the reproductive stage	IR64 × Cabacu	SNP	Recombinant inbred line	1	[55]
Yield under reproductive stage stress over seasons	Swarna × WAB	SSR	Backcross inbred line	1	[56]
Heritability for grain yield	CT9993 × IR62266	AFLP	Doubled haploid lines	1	[57]
Grain yield under severe lowland drought over environments	R77298 × Sabitri,	SSR	BC1 derived	1	[58]
Grain yield over years and location	Apo/2 × Swarna	SSR	Recombinant inbred lines	1	[59]
Yield at reproductive stage over environments	Two populations	SSR	Bulk-segregant analysis	2	[60]
Morphological and physiological traits	IR64 × Azucena	RFLP	Doubled haploid Lines	15	[61]
Dehydration avoidance	Bala × Azucena	RFLP, AFLP & SSR	Recombinant inbred lines	17	[62]
Osmotic adjustment and Dehydration tolerance	CO39 × Moroberekan	RFLP	Recombinant inbred line	1	[63]
Osmotic adjustment	CT9993 × IR62266	RFLP, AFLP & SSR	Doubled haploid line	5	[64]

**Table 2:** Drought tolerant gene that has been tested in rice.

Gene Action	Gene	Promoter	Transformation	Phenotype	References
**Genes Encoding Enzymes That Synthesize Osmotic and Other Protectants**					
Arginine decarboxylase	*ADC*	*CaMV35S*	Biolistic	Reduction in chlorophyll loss under water deficiency	[79]
Polyamine synthesis	*ADC*	*Ubi-1*	*Agrobacterium*	Improved drought tolerance by producing higher levels of putrescine and spermine synthesis.	[16]
abscisic acid Metabolism	*CaMV35SP*	*DSM2*	*Agrobacterium*	Oxidative and drought stress resistance and increase of the xanthophylls and non-photochemical quenching.	[80]
Amino acid metabolism	*OsOAT*	*Ubi1*	*Agrobacterium*	Improve drought tolerance and increase seed setting	[77]
Reactive oxygen species scavenging	*OsSRO1c*	*Ubi1*	*Agrobacterium*	Oxidative stress tolerance and stomata closure regulation	[81]
Protoporphyrinogen oxidase	*PPO*	*-*	*Agrobacterium*	Less oxidative damage, and drought tolerance	[82]
Trehalose synthesis	*OsTPS1*	*Actin1*	*Agrobacterium*	Tolerance of rice seedling to drought, cold, and high salinity	[83]
Trehalose synthesis	*TPSP*	*Ubi1*	*Agrobacterium*	Cold, salt and drought tolerance expressed by chlorophyll fluorescence	[78]
Proline synthesis	*P5CS*	*Act1*	*Agrobacterium*	Resistance to water and salinity stress	[84]
Proline synthesis	*P5CS*	*AIPC*	Biolistic	Increased biomass production under salinity and drought stresses	[76]
**Late Embryogenesis Abundant (LEA) Related Genes**					
LEA protein gene	*HVA1*	*Actin1*	*Agrobacterium*	Cell membrane stability, higher leaf relative water content (RWC) and increase in growth under drought stress.	[73]
	*HVA1*	*Actin1*	*Agrobacterium*	Drought and salinity tolerance	[85]
	*HVA1*	*Actin 1*	Biolistic	Increased drought tolerance and salinity stress.	[86]
	*OsLEA3-1*	*rice LEA3-1*	*Agrobacterium*	Drought resistance for grain yield under field conditions	[71]
	*OsLEA3-2*	*CaMV35S*	*Agrobacterium*	Drought resistance and increase grain per panicle	[70]
**Various Regulatory Genes**					
Transcription factor	*ABF3*	*Ubi1*	*Agrobacterium*	Improved salinity and drought tolerance	[87]
	*AP37*	*OsCc1*	*Agrobacterium*	Improve growth performance under drought stress	[88]
	*OsbZIP23*	*Ubi1*	*Agrobacterium*	Wide spectrum to salt and drought tolerance and improvement in yield.	[89]
	*OsbZIP72*	*CaMV35S*	*Agrobacterium*	Drought resistance and ABA sensitivity	[90]
	*DREB1 or OsDREB1*	*CaMV 35S*	*Agrobacterium*	Tolerance to water deficient, low-temperature and high-salt stresses	[91]
	*DREB2*	*rd29A*	*Agrobacterium*	Improve grain yield under drought stress	[92]
	*HvCBF4*	*Ubi1*	*Agrobacterium*	Tolerance to drought, high-salinity, and low-temperature.	[93]
Harpin protein	*Hrf1*	*CaMV 35S*	*Agrobacterium*	Drought resistance through ABA signalling and antioxidants, and stomata closure regulation	[94]
Jasmonate and ethylene-responsive factor 1	*JERF1*	*CaMV35S*	*Agrobacterium*	Drought resistance	[95]
Ethylene-responsive factor 1	*TSRF1*	*-*	*Agrobacterium*	Enhances the osmotic and drought tolerance	[96]
RING finger protein	*OsCOIN*	*CaMV35S*	*Agrobacterium*	Cold, salt and drought tolerance	[97]
Stress/zinc finger protein	*OsiSAP8*	*-*	*Agrobacterium*	Tolerance to salt, drought and cold stress	[98]
Protein degradation (E3 ubiquitin ligase)	*OsRDCP1*	*CaMV35S*	*Agrobacterium*	Improved tolerance to drought stress	[99]
	*OsSDIR1*	*Ubi1*	*Agrobacterium*	Stomata regulation under drought stress	[100]
